# Structural insights into the inhibited states of the Mer receptor tyrosine kinase

**DOI:** 10.1016/j.jsb.2008.10.003

**Published:** 2009-02

**Authors:** Xudong Huang, Patrick Finerty, John R. Walker, Christine Butler-Cole, Masoud Vedadi, Matthieu Schapira, Sirlester A. Parker, Benjamin E. Turk, Debra A. Thompson, Sirano Dhe-Paganon

**Affiliations:** a Structural Genomics Consortium, University of Toronto, Toronto, Ont., Canada; b Department of Physiology, University of Toronto, Toronto, Ont., Canada; c Department of Pharmacology, University of Toronto, Toronto, Ont., Canada; d Department of Pharmacology, Yale University School of Medicine, New Haven, CT 06520, USA; e Department of Ophthalmology and Visual Sciences, University of Michigan Medical School, W.K. Kellogg Eye Center, Ann Arbor, MI 48105-0714, USA; f Department of Biological Chemistry, University of Michigan Medical School, W.K. Kellogg Eye Center, Ann Arbor, MI 48105-0714, USA

**Keywords:** Mer, MerTK, Kinase, Structure, Autoinhibition, Screen, Chemical probe

## Abstract

The mammalian ortholog of the retroviral oncogene v-Eyk, and a receptor tyrosine kinase upstream of antiapoptotic and transforming signals, Mer (MerTK) is a mediator of the phagocytic process, being involved in retinal and immune cell clearance and platelet aggregation. Mer knockout mice are viable and are protected from epinephrine-induced pulmonary thromboembolism and ferric chloride-induced thrombosis. Mer overexpression, on the other hand, is associated with numerous carcinomas. Although Mer adaptor proteins and signaling pathways have been identified, it remains unclear how Mer initiates phagocytosis. When bound to its nucleotide cofactor, the high-resolution structure of Mer shows an autoinhibited αC-Glu-out conformation with insertion of an activation loop residue into the active site. Mer complexed with compound-52 (C52: 2-(2-hydroxyethylamino)-6-(3-chloroanilino)-9-isopropylpurine), a ligand identified from a focused library, retains its DFG-Asp-in and αC-Glu-out conformation, but acquires other conformational changes. The αC helix and DFGL region is closer to the hinge region and the ethanolamine moiety of C52 binds in the groove formed between Leu593 and Val601 of the P-loop, causing a compression of the active site pocket. These conformational states reveal the mechanisms of autoinhibition, the pathophysiological basis of disease-causing mutations, and a platform for the development of chemical probes.

## 1 Introduction

Mer is a transmembrane tyrosine receptor kinase with extracellular immunoglobulin and fibronectin-like domains that recognize ligands such as growth arrest specific 6 (Gas6) and protein S (Hafizi and Dahlback, 2006a). Ligand-binding leads to autophosphorylation of activation loop tyrosine (Tyr749, Tyr753 and Tyr754) (Ling et al., 1996), recruitment of adaptor proteins (Grb2, LimD4, and Vav1) (Colland et al., 2004; Georgescu et al., 1999; Ling and Kung, 1995; Mahajan and Earp, 2003), and activation of downstream enzymes (PI3K, MAPK, and GTPase) (Chen et al., 1997; Ling and Kung, 1995). Mer behaves as a proto-oncogene, having roles in cellular transformation of NIH/3T3 and B-lymphocytes cells (Georgescu et al., 1999; Ling and Kung, 1995). Its expression and activity is elevated in various human cancers including pituitary adenomas (Evans et al., 2001), mantle cell lymphomas (Ek et al., 2002), and T-cell acute lymphoblastic leukemia (Graham et al., 2006). Transgenic mice that overexpress Mer in lymphocytes and thymocytes developed lymphoblastic leukemia/lymphoma (Keating et al., 2006). Mice lacking either Gas6 or Mer have impaired platelet aggregation in vitro and decreased stability of clots in vivo (Angelillo-Scherrer et al., 2001; Chen et al., 2004). And Mer mediates phagocytic processes that occur in monocytes, macrophages, and retinal pigment epithelial cells. Mutations in the *MerTK* gene have been shown to disrupt retinal pigment epithelial phagocytosis in rats and mice (D’Cruz et al., 2000; Duncan et al., 2003), and result in retinitis pigmentosa in patients (Gal et al., 2000). Mer-deficient mice also exhibited impaired clearance of apoptotic thymocytes (Scott et al., 2001). Although each member of the Mer subfamily, including Axl and Sky, functions in regulating cell proliferation and platelet aggregation, it appears that only Mer is involved in provoking phagocytosis. Given the role of Mer in the pathophysiology of thrombosis and tumorigenesis, including acute lymphoblastic leukemia, inhibition of Mer may be an option for therapeutic intervention of these diseases.

Given the role of Mer in the pathophysiology of thrombosis and tumorigenesis, including acute lymphoblastic leukemia, inhibition of Mer may be an option for therapeutic intervention in these diseases. A soluble form of Mer that acts as an antagonist decreased platelet aggregation in vitro and prevented fatal collagen/epinephrine-induced thromboembolism in mice (Sather et al., 2007). And Mer or Gas6 knockout mice were protected from collagen/epinephrine-induced pulmonary thromboembolism and ferric chloride-induced thrombosis (Angelillo-Scherrer et al., 2001; Chen et al., 2004). So far, no specific small molecule inhibitors for Mer have been reported. Recent successes in the treatment of chronic myelogenous leukemia with Imatinib, a small molecule that targets the constitutively active tyrosine kinase BCR-ABL (Druker et al., 1996; Savage and Antman, 2002), has encouraged us to study the biochemical properties of Mer and search for chemical probes despite the challenges posed by major overall similarity in the ATP binding sites of protein kinases.

In contrast to the amino-terminal lobes, the helical carboxyl-terminal lobe of protein kinases is considerably more conserved in terms of both primary and tertiary sequence. While the carboxy-terminal lobe evolved to contain important determinant for substrate binding, the amino-terminal lobe of protein kinases contain many determinants that control the reaction cycle. The amino-terminal lobe is composed of a twisted five-stranded β-sheet with a closely associated alpha-helix (αC) running the length of one side of the β-barrel-like substructure. The pocket formed between the carboxy- and amino-terminal lobes is the site of binding of ATP as well as many small molecule inhibitors or probes. We have begun to investigate the structural characteristics of the Mer kinase domain and its active site with structures of various ligands. The amino-terminal lobe adopts an orientation with the DFG-Asp in the “in” position and the αC-Glu in the “out” position. We characterized determinants of Mer inhibition by screening small molecule inhibitors and elucidated the structural interaction between an inhibitor and the Mer kinase domain. Our studies represent the first solved structure of the intracellular kinase domain of a member of the Axl/Mer/Sky RTK phylogenetic branch, provide insight into the mechanism of inhibition of Mer tyrosine kinase activity, and set a platform for future studies of Mer sequence variants in retinitis pigmentosa.

## 2 Materials and methods

### 2.1 RTK expression and purification

The Mer kinase domain (residues 588–855) and the catalytic domain of human protein tyrosine phosphatase PTPN1 (1–283 residues) were cloned together as a bicistron into a bacterial expression vector pET28-LIC. The kinase ORF #1 contained an N-terminal His tag and thrombin cleavage site. The untagged phosphatase was ORF #2. The intergenic sequence contained a ribosomal binding site (CTCGACGGAGGAATAATCAT). Plasmids were transformed into BL21(DE3) cells, grown in TB media at 37 °C using an aeration system (LEX), and induced at 15 °C with 100 μM IPTG. Cell pellets were harvested after overnight bubbling at 15 °C and stored at −80 °C.

The Mer protein was purified in two chromatographic steps: immobilized metal affinity chromatography on a Talon resin (Qiagen) and gel filtration. Cell pellets from 4 L culture were homogenized in 100 ml lysis buffer containing 50 mM Tris–HCl pH 8.0, 500 mM NaCl, 5% glycerol, 1 mM β-mercaptoethanol, 2 mM imidazole, and lysed using a microfluidizer at 18,000 psi (Microfluidics). The lysed cells were centrifuged at 69,000*g* for 1 h. The supernatant was incubated with 5.0 ml 50% Talon resin for 1 h at 4 °C, and then washed two times with 25 ml washing buffer (50 mM Tris–HCl pH 8.0, 500 mM NaCl, 5% glycerol, 1 mM β-mercaptoethanol, 10 mM imidazole). The protein was eluted with 7 ml elution buffer (50 mM Tris–HCl pH 8.0, 500 mM NaCl, 5% glycerol, 1 mM β-mercaptoethanol, 200 mM imidazole). The eluent was further purified by the gel filtration AKTAxpress system on a 16/65 Superdex 200 column (GE Healthcare) pre-equilibrated with the gel filtration buffer (20 mM Tris, pH 8.0, 500 mM NaCl, 5% glycerol, 2 mM β-mercaptoethanol). The yield of 7.3 mg/L of bacterial culture; purified protein was frozen in liquid nitrogen and stored at −80 °C. Autophosphorylation of Mer was performed at room temperature for 3 h with 10 mM ATP (Sigma, A7699) and 10 mM MgCl_2_ in gel filtration buffer (20 mM Tris, pH 8.0, 500 mM NaCl, 5% glycerol, and 2 mM β-mercaptoethanol).

### 2.2 Positional scanning peptide screens

Mer phosphorylation specificity was determined by screening a library of 198 peptide mixtures. Each peptide had the general sequence G-A-X-X-X-X-X-Y-X-X-X-X-A-G-K-K(biotin), in which 8 of the 9 positions indicated as X were equimolar mixtures of the 18 amino acids, excluding tyrosine and cysteine. The remaining X position was fixed as one of the 20 amino acids or as phosphothreonine or phosphotyrosine. Peptides (50 μM) were incubated in sealed multiwell plates for 2 h at 30 °C with phosphorylated Mer (80 ng/ml) in 50 mM Tris, pH 7.5, 10 mM MgCl_2_, 1 mM DTT, 0.1% Tween-20, with 50 μM [^33^P]-γ-ATP (0.03 μCi/μl). Aliquots of the reactions were then spotted onto a streptavidin coated membrane, which was washed, dried and exposed to a phosphor screen as described (Turk et al., 2006).

### 2.3 Kinase Inhibitor library screening

A library of kinase inhibitors was screened using differential scanning fluorimetry with a FluoDia T70 (PTI) according to the outlined protocol (Niesen et al., 2007). The library consisted of 157 compounds at 50 μM concentration in 100 mM Hepes pH 7.5, 150 mM NaCl.

### 2.4 Docking simulations

Flexible compounds were initially docked in a rigid grid representation of the receptor with Glide XP (Schrodinger LLC). In the case of staurosporine and compound-52, minor side-chain rearrangements were necessary to accommodate the ligand, and ICM (Molsoft LLC) was used to minimize in the internal coordinate space by a Monte Carlo simulation the energy of a system comprised of the flexible and mobile ligand and flexible receptor side-chains within 7 Å of the active site (Totrov and Abagyan, 1997).

### 2.5 Protein kinase assays

Kinase activity assays, including those used for kinetics measurements, were performed using the ADP Quest Assay (DiscoveRx) and following the protocol for kinetic mode provided by the manufacturer. Kinase activity was monitored using a BioTek Synergy 2 plate reader operating in kinetic mode and fluorescence intensity measurements (540 nm excitation and 620 nm emission) were made once per minute. Mer proteins used in the kinetic assays were present at 25 nM concentration, the ATP concentration ranged from 10 to 200 μM, and the peptide concentration ranged from 0.1 to 2 mM. Fluorescence intensity values were converted to moles of ADP using a standard curve prepared as described in the ADP Quest Assay manual and the correlation between ADP concentration and fluorescence intensity was linear over the range of values obtained in these experiments. Dual-substrate experiments that were used to calculate kinetic parameters were performed in duplicate. Kinase reaction rates were calculated using linear regression with data from 5 to 11 min of the reaction, a time over which the reaction generally produced linear data. The background reaction rate, resulting from ADP contamination of the ATP stock solution, was measured in a reaction lacking enzyme or substrate and subtracted from the experimental rates. Kinetics parameters were obtained by fitting the rates using the two-substrate format in the Enzyme Kinetics module of SigmaPlot v9.01 and the data were best fit with a Random Bi–Bi Sequential model. The time-response comparison of Mer (543.864) was performed in the same manner as the kinetics experiments except that only a single concentration of ATP (250 μM) and peptide (2 mM) were employed. Mer proteins were used at 25 nM. IC50 experiments were performed using the ADP Quest Assay in kinetics mode. All compounds assayed in the experiments were determined to be compatible with the assay in separate experiments (data not shown). ATP-treated Mer was at 25 nM, ATP was 250 μM, and the peptide substrate was at 2 mM. Depending on the experiment, either four or five different concentrations of each inhibitor were tested and each set of experiments were performed in duplicate.

### 2.6 Crystallization, data collection, phasing, and refinement

All crystals were grown at 14 °C using the hanging-drop vapor-diffusion method. Mer-ANP complex crystals were grown by mixing 2 μl Mer solution (35 mg/ml Mer (571–864), 2.5 mM ANP, 10 mM MgCl_2_) and 2 μl reservoir solution (100 mM Tris–HCl pH 8.5, 200 mM MgCl_2_, 29% PEG 400). Mer-ADP crystals were obtained by pre-incubating 38 mg/ml Mer protein with 2.5 mM ATP and 10 mM MgCl_2_ for 3 h at room temperature and grown by mixing 2 μl with 2 μl reservoir solution as above. Compound-52 (50 mM in DMSO) was added to 8 mg/ml Mer protein to the final concentration of 2.5 mM, rocked at 4 °C overnight, and further concentrated to about 35 mg/ml. Crystals were grown by mixing 2 μl Mer and 2 μl reservoir solution (100 mM Tris–HCl pH 8.5, 3.64 M NaCl). Crystals were frozen in a cryoprotectant composed of 9% sucrose (wt/vol), 2% glucose (wt/vol), 8% glycerol (vol/vol), and 8% ethylene glycol (vol/vol) in reservoir buffer. Data for the ANP co-crystal structure was collected on a Rigaku FR-E equipped with a RAXIS-4++ detector. The data were processed with HKL-2000 (Minor et al., 2002; Otwinowski and Minor, 1997), and then input into the program PHASER (Read, 2001) using the pdb entry 2G15 as the molecular replacement search model. Iterative manual model building using the graphics program O (Jones et al., 1991), density modification and automatic model building using RESOLVE (Terwilliger, 2000), and TLS and restrained refinement using Refmac 5.2 (1994) resulted in a model with a working *R* value of 0.20 and a free *R* value of 0.27 for the resolution range from 2.4 to 24.8 Å. Two molecules of the MerTK catalytic domain are present in the asymmetric unit. Residues 552–574, 596–597, 622–632, 658–664, 745–762, and 862–864 in chain A, as well as residues 552–574, 596–598, 622–632, 658–664, 745–762, 862–864 in chain B were not located in the experiment.

Data for the compound-52 co-crystal structure was collected at the Argonne Photon Source IMCA-CAT beamline 17ID, and processed using HKL-2000. Chain A from the ANP co-crystal structure was used as the molecular replacement model for this structure. Manual building of the model was carried out using the graphics program Coot (Emsley and Cowtan, 2004) and TLS and restrained refinement using Refmac 5.2 resulted in a model with a working *R* value of 0.27 and a *R* free of 0.30 for data from 47.3 to 2.8 Å. Four molecules of the Mer catalytic domain are present in the asymmetric unit. Tight NCS restraints were used in the refinement of this structure. Residues from chain A 552–574, 623–666, 746–762, 864; chain B 552–574, 596, 625–630, 660–666, 746–762, 864; chain C 552–574, 623–630, 660, 663–666, 746–762, 864; and chain D 552–574, 623–630, 662–666, 746–762 and 864 were not located in the experiment.

Data for the ADP co-crystal structure was collected at the F-1 beamline at the Cornell High Energy Synchrotron Source, and processed using HKL-2000. Chain A from the AMP-PNP co-crystal structure was used as the molecular replacement model for this structure. Autobuilding of the structure and location of water molecules was carried out using the program ARP/wARP (Perrakis et al., 1999), followed by iterative manual model building using the graphics program Coot, and TLS and restrained refinement using Refmac 5.2. For each of the three structures the initial TLS parameters were generated using the TLSMD web server (Painter and Merritt, 2006). Two molecules of the MerTK catalytic domain are present in the asymmetric unit. Residues from chain A 552–574, 596, 622–624, 659–665, 746–761, 864; and chain B 552–574, 745–762, and 864 were not located in the experiment. Further details of data collection and refinement can be found in the Table 1.

## 3 Results and discussion

### 3.1 Overall structures

**Fig. 1:**
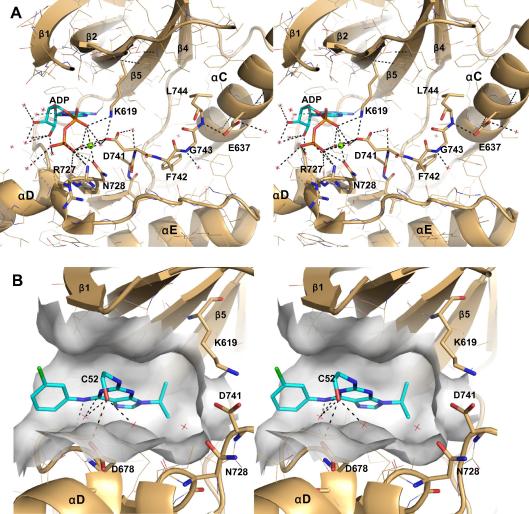
Active site structure and complex with compound-52. (A) A close-up, stereoscopic view of the active site of Mer (ADP bound) is shown in Pymol cartoon format, colored light brown. ADP is colored cyan and shown in stick format. The DGF loop and αC-Glu are also shown in stick format and labeled. Water molecules within 6 Å of the nucleotide and catalytic residues are shown as red stars, and the magnesium ion as a green sphere. Hydrogen bonds with distances less than 3.2 Å are shown as black, dashed lines. (B) Stereoscopic view of the ligand-binding pocket of Mer. A carved, surface representation of the pocket formed upon compound-52 binding is shown colored transparent-gray. Surrounding, catalytic side-chains are shown in stick format; waters and hydrogen bonds to the ligand are shown as above.

To characterize the active site and regulatory mechanisms of Mer and advance rational approaches toward the development of chemical probes, crystal structures of its kinase domain with nucleotide ligands ADP and ANP and the inhibitor compound-52 (C52) were determined (Table 1). These structures had good electron density for most residues and revealed the canonical protein kinase fold: β-stranded amino-terminal and α-helical carboxy-terminal lobes. The ADP and ANP nucleotide structures superimposed very well with an RMSD of 0.11 Å. These structures show that the DFG-Asp motif is in the “in” position, with Phe742 buried in its canonical pocket, forming van der Waals contacts with residues in the αC helix, αC-β4 loop, β6-strand, and αE helix (Fig. 1A). The DFG-Asp741 is poised in a catalytically competent state; its carboxyl group is involved in hydrogen bonds with (1) a chelated magnesium ion and its coordinated water molecules, (2) the amino group of the catalytic lysine (Lys619), and (3) the amide group of the catalytic Asn728. In addition, the carboxyl group is hydrogen bonded to a water molecule that is trapped in the core of the active site, between but not hydrogen-bonded to the DFG-Phe742, Ala740, Leu671, and the nucleotide. The magnesium ion is coordinated to the α- and β-phosphoryl oxygens, two axial water molecules, the Asn728 side-chain, and the DFG-Asp741 carboxyl group, consistent with a typical protein kinase catalytically competent state. However, αC-Glu (Glu637), which is a critical residue for catalytic turnover, is in the αC-Glu-out conformation (Fig. 1A). Although mostly solvent-exposed, one oxygen atom of its carboxyl group hydrogen-bonds with the backbone amide of Leu744, the first and only residue of the activation loop that is ordered in our structures. The side-chain of Leu744 is buried in the interlobe pocket, above DFG-Phe742 and between the αC helix and the β4- and β5-strands; it is also protected from the solvent by the side-chain of the αC-Glu637. This residue is conserved among Mer paralogs and its aliphatic nature is conserved among all receptor tyrosine kinases; the analogous residues of EGFR paralogs have been implicated in regulatory transitions, stabilizing the inhibited, αC-Glu-out conformation; mutagenesis of this residue to arginine leads to activation of EFGR1 (Zhang et al., 2006). Mer was superimposed with other DFG-Asp-in/αC-Glu-out structures, including those from Abl, Src, Egfr, Chk, and Met kinases, showing that the orientations of αC helices relative to the rest of the β-lobe domain were similar. Also conserved in these inhibited states is an aliphatic residue immediately following DFG that inserts into the space generated by the outward movement of the αC helix. The αC-Glu-out conformation represents one of the most familiar inhibited tyrosine kinase states, and it likely evolved as an efficient way to minimize inappropriate activation of the receptor under basal, unstimulated conditions.

**Supplementary Fig. 1:**
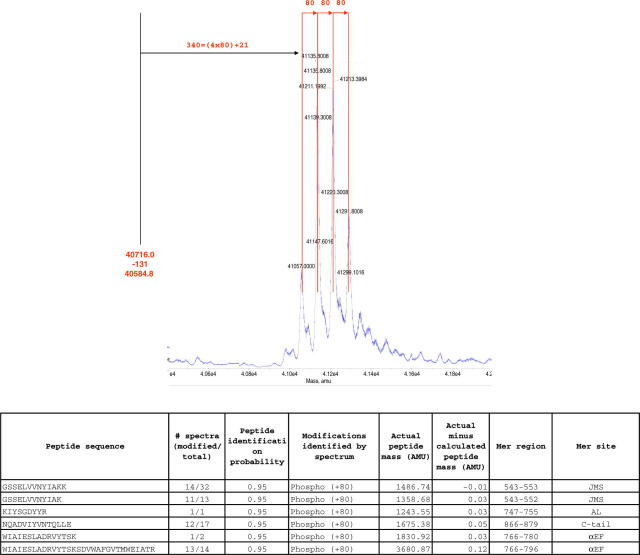


**Supplementary Fig. 2:**
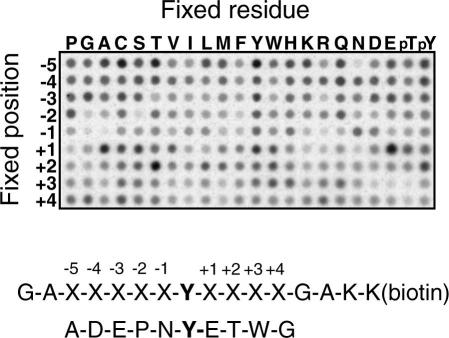


Mer contains a number of tyrosine residues in the juxtamembrane and C-terminal tail that in addition to being adaptor protein binding sites (Georgescu et al., 1999) could also regulate catalytic efficiency. To attempt to characterize how upstream signals induce activation of the Mer kinase enzyme, we conducted biochemical analyses, focusing on the role of the juxtamembrane segment (JMS) because this region of the protein contains a rare variant (E540K) associated with retinal disease (Tada et al., 2006). In order to explore the possibility that Mer is autoregulated by an autophosphorylation event of its juxtamembrane region (which contains a single tyrosine (Tyr549)), we first showed by mass-spectrometry (infusion electrospray MS and LCMS/MS) that the Mer JMS tyrosine (Tyr549) is a potential autophosphorylation site. The phosphatase-coexpressed kinase, which was devoid of phosphorylation sites, could be modified by treatment with ATP, and supports the possibility of a post-translational regulatory event (Supplementary Fig. 1). To test if unphosphorylated Tyr549 is inhibitory, in a manner analogous to the JMS tyrosine residues of Eph receptors (Davis et al., 2008; Wiesner et al., 2006; Wybenga-Groot et al., 2001), we followed the reaction using dephosphorylated forms of the JMS-containing kinase constructs (Binns et al., 2000). For these studies, a 9 residue peptide substrate was developed using a positional scanning peptide library (Hutti et al., 2004). The resulting phosphorylation profile suggests low side-chain specificity in all positions −5 through +4, with only minor preferences, for example, at the +1 position (favorable glutamate) and the +3 position (negative selection against acidic residues) (Supplementary Fig. 2). Spot intensities were determined and ranked, generating the optimized peptide: ADEPNYETWG. The enzyme had robust catalytic activity with this substrate, initially suggesting that the inhibited, crystalline state is not a dominant or stable species in solution. When compared to unphosphorylated wild-type kinase, the ATP-pretreated wild-type kinase had similar initial rates (data not shown), suggestion either that the JMS is not involved in regulation or that phosphorylation of the JMS is faster than our assay setup, a scenario reminiscent of the Eph kinase system (Binns et al., 2000). To trap potential inhibitory species, we mutated the single juxtamembrane segment tyrosine (Tyr549) to phenylalanine. We found that instead of inhibiting the enzyme, as might be suspected in a scenario such as the Eph kinase (Wybenga-Groot et al., 2001), we found that this mutation caused mild activation of the enzyme. At saturating ATP concentrations (200 μM), wild-type kinase catalyzed phosphorylation of the optimized peptide with a *k*_cat_/*K*_m_ of 0.0010 ± 0.0001 M^−1^ s^−1^ and the Y549F mutant with a *k*_cat_/*K*_m_ of 0.0051 ± 0.0002 M^−1^ s^−1^. That the mutation potentiated the enzyme suggests that the juxtamembrane segment is not likely to have an autoinhibitory role analogous to Eph kinases. It remains possible that the juxtamembrane is involved in a phosphorylation-independent, autoactivation mechanism analogous to that of EGFR (Jeffrey et al., 1995; Zhang et al., 2006). Although size-exclusion chromatography did not provide evidence for JMS-dependant dimerization (data not shown), the Mer JMS could accelerate autoactivation through transient homophilic interactions in a manner consistent with ligand-dependant receptor dimerization and activation (Hafizi and Dahlback, 2006a,b; Hafizi et al., 2002; Taylor et al., 1995). The function of juxtamembrane Tyr549 and its possible phosphorylation remains unknown.

### 3.2 Mutations in retinitis pigmentosa

**Fig. 2:**
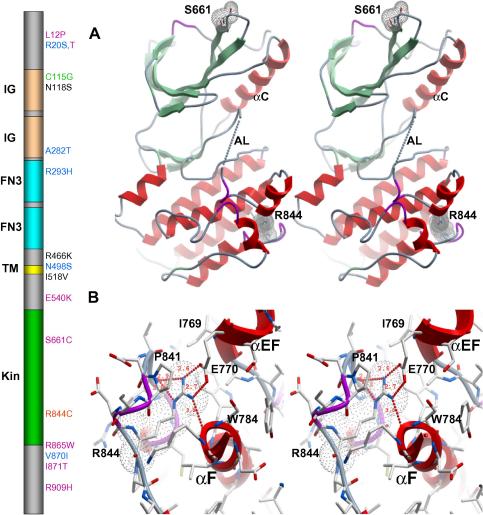
Coding sequence variants of human Mer. Schematic representation of the primary structure of human Mer showing missense variants identified in retinitis pigmentosa patients is shown to the left of this figure. IG, immunoglobulin domain; FN3, fibronectin type 3 domain; TM, transmembrane segment; Kin, kinase domain. Missense variants are colored as follows: black, common variant; blue, rare variant with similar frequency in patients and controls; purple, rare variant detected in patients and not controls; green, rare variant with proposed association with retinitis pigmentosa; red, rare variant with in vitro loss-of-function and associated with retinitis pigmentosa. (A) Stereoview of the Mer kinase domain in ribbon format with red α helices, green β-strands, and purple 3_10_ helices and Ser661 and Arg844 are displayed in dot envelope representation using Molsoft ICM. Also labeled are αC and AL as a dotted line. (B) Stereoview of Arg844 in stick format, buried in the C-terminal lobe, forming a salt bridge with Glu770 and hydrogen bonds with the backbone atoms of Arg838 and Lys780, also in stick format. Indicated as dashed lines are all hydrogen bonds formed by the side-chain of Arg844.

Mutation screening of the *MERTK* gene in retinal dystrophy patients has identified a number of disease-associated changes, including point mutations and small rearrangements predicting premature protein truncation and splicing defects (Ebermann et al., 2007; Gal et al., 2000; McHenry et al., 2004; Tada et al., 2006; Tschernutter et al., 2006). Several missense variants were also identified, representing both frequent and rare polymorphisms, some of which are located in the Mer intracellular region: E540K, S661C, R844C, R865W, I871T, and R909H (Fig. 2, left). To date, there is clear evidence (genetic and/or biochemical) for disease-association of only one Mer missense change: R844C. The Cys844 variant segregated with retinitis pigmentosa in the affected family, and the corresponding recombinant protein exhibited decreased expression and tyrosine kinase activity, and increased protein turnover (McHenry et al., 2004). Arg844 projects its guanidinium group into the core of the C-terminal lobe, forming a buried salt bridge with the carboxyl group of Glu770 and hydrogen-bonding with backbone atoms of Lys780 and Arg838 (Fig. 2B). As part of the “APE” αEF helix, Glu770 represents an anchor point for the activation loop, as it is intimately associated with proper substrate binding through the P + 1 and αEF-αF loops, helping to shape the platform on which substrates and activation loop folds (Nolen et al., 2004). This E:R ion pair is conserved among many kinases, and its mutation to cysteine could lead to protein destabilization, consistent with increased protein turnover in vitro and associated pathophysiology in vivo.

**Supplementary Fig. 3:**
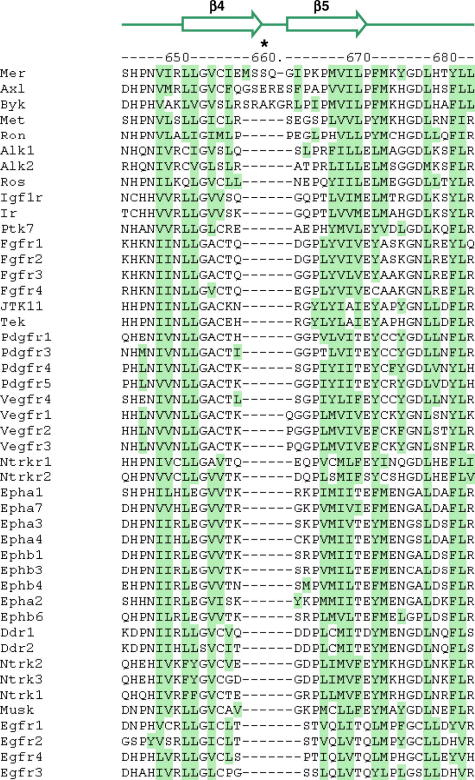


Although other rare Mer coding variants have been detected only in retinal dystrophy patients, their effect on function has not yet been established. The rare variant S661C is also located in the Mer kinase domain. However, our structure shows that Ser661 is completely solvent exposed in the β4–β5 loop (Fig. 2A), and interestingly it is one of six residues in the β4–β5 loop unique to this phylogenetic branch (Supplementary Fig. 3), the role of which is unknown. Thus, it appears that the S661C substitution would not radically affect function, consistent with it being a rare variant with unknown disease-association. Nevertheless, its location in a unique insertion sequence may point to a role in catalysis. Taken together, our Mer structure will be useful for predicting whether amino acid differences corresponding to human coding variants in the intracellular domain are likely to perturb critical functional interactions, and for planning future studies to evaluate involvement in the pathogenesis of retinitis pigmentosa.

### 3.3 Kinase inhibitors

**Supplementary Fig. 4:**
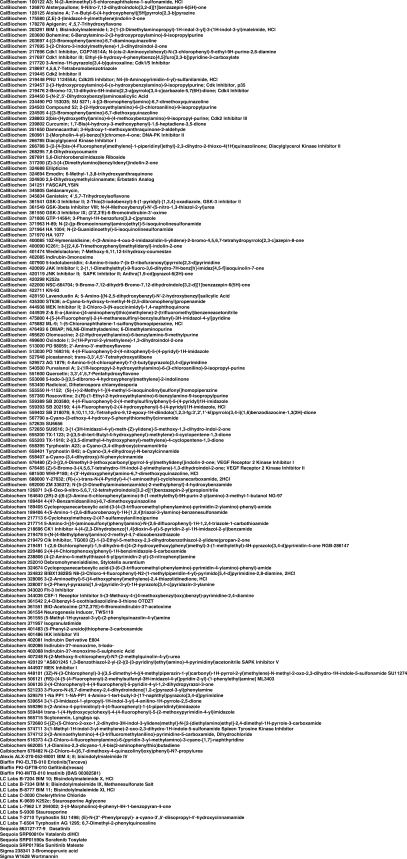


**Fig. 3:**
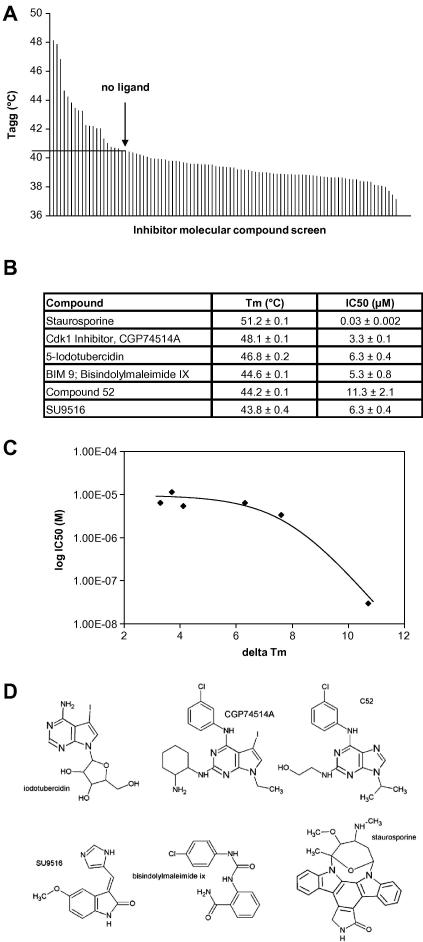
Kinase library screen and validation. (A) Average and standard deviation of the melting temperature of untreated Mer kinase domain, as determined by differential scanning fluorimetry, in the presence of compounds, and sorted by value. The Tm of the protein without ligand is highlighted (40.5 °C). (B) Six compounds that induced the highest shifts in the *T*_m_ of Mer are shown, along with the *T*_m_ of Mer in the presence of each compound as well as IC50 values measured for each compound according to the methods section. *T*_m_ values are an average of two measurements made on the same plate. (C) Correlation plot between IC50 values and delta-*T*_m_. (D) Chemical structures and names of inhibitors of Mer.

Because Mer is associated with tumorigenesis and thrombosis, chemical probes that target Mer functions may be scientifically and therapeutically useful. To generate a preliminary ligand-specificity map of the active site, a 157-compound kinase inhibitor library (Supplementary Fig. 4) was screened using differential scanning fluorimetry (Niesen et al., 2007; Senisterra et al., 2006). ATP-pre-treated Mer at 10 μM was pre-incubated with compounds at 50 μM and subjected to thermal denaturation. Six compounds stabilized Mer with a value greater than 4 °C compared with the average aggregation temperature of 38.5 ± 0.2 °C (Fig. 3A and D). All hits were validated with dose response analysis using either DSF or differential static light scattering (DSLS). Additionally, the compounds were used in inhibition assays, in conjunction with the ADP Quest Kinase assay kit using the optimized peptide substrate, to obtain IC50 values with a correlation with delta-*T*_m_ (Fig. 3B and C). Ligand hits can be subdivided into two general scaffold classes: (1) purine derivatives and (2) staurosporine and other pyrrolinone or maleimide-based compounds (Fig. 3D, top and bottom, respectively).

To determine the mode of binding of these compounds, inhibitors were co-crystallized, and a complex structure was determined with C52, a ligand developed from combinatorial synthesis of 2,6,9-trisubstituted purines (Gray et al., 1998). Related compounds purvalanol B and roscovitine were studied previously in complex with Src and Cdk2 (Breitenlechner et al., 2005; De Azevedo et al., 1997; Gray et al., 1998). C52 bound co-planar with ATP of the Mer–ATP complex, occupying the adenine and sugar pockets (Fig. 1B) and having good shape-complimentarity to this region of the pocket; its isoproply group fills the space formed between Ala617, Lys619, Met730, Asp741, and Leu671 and its ethanolamine moiety binds in the groove formed between Leu593 and Val601. The hydroxyl group of C52 hydrogen-bonds to the carboxylate of Asp678 and the carbonyl oxygen of Arg727. Compared with the nucleotide-bound complex, the N-terminal lobe of the C52 complex is rotated (about 5°) relative to and is more closely associated with the C-terminal, helical lobe. For example, the distance between the tertiary carbon atom of Val601 and the sulfur atom of Met730 is 1.0 Å less in the C52 complex structure. In addition, hydrogen bonds are present between (1) the purine N7 nitrogen and the backbone nitrogen of Met674, (2) the ligand’s aniline nitrogen and the carbonyl of Met674, and (3) the ligand and the carboxylic group of Asp678; one via a water molecule. Complementary, van der Waals interactions also form between the chloro-phenyl ring of C52 and backbone atoms of the hinge region (residues 674–676). Retaining its DFG-Asp-in and αC-Glu-out conformation, C52 draws the entire N-lobe, including αC, towards the hinge region (674–676), causing relocation of the aliphatic DFGL-Leu744.

**Fig. 4:**
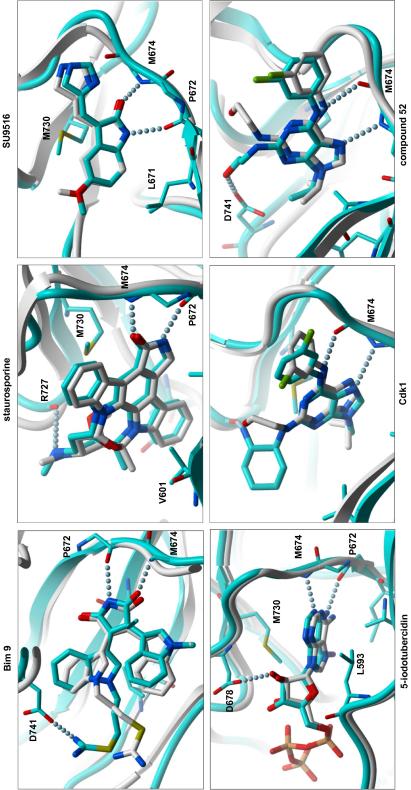
Docking of inhibitors in Mer. All Mer inhibitors, including compound-52, were docked using Glide and ICM into the active site of the high-resolution, Mer structure. Docked complexes are displayed in cyan cartoon and stick formats. Aligned and colored in gray are complex structures of MAPKKK5-staurosporine (2CLQ), CDK2-SU9516 (1PF8), CAMK2G-BIM-9 (2V7O), Mer-ANP (2P0C), and Mer-C52 (3BPR).

In order to better understand the specificity of the pocket, the remaining ligand hits were docked to the ATP site of our high-resolution Mer crystal structure bound to ATP with Glide (Schrodinger) and ICM (Molsoft LLC). The pyrrolinone group of staurosporine and SU9516, and maleimide group of BIM-9 make a canonical dual hydrogen bond with the backbone nitrogen of M674 and carbonyl of P672; the docked conformations are in agreement with co-crystal structures of staurosporine, SU9516 and BIM-9 bound to other kinases (2CLQ, 1PF8, 2V7O, respectively) (Fig. 4). The docked conformation of C52 recapitulates our co-crystal structure, but the hydroxyethanolamine tail hydrogen-bonds to the carboxylate group of Asp741 instead of Asp678. The close analog CDK1 inhibitor is posed in a very similar way. And, 5-iodotubercidine mimics the binding of adenosine in our ATP co-crystal, with the additional iodide deeply buried in a hydrophobic cavity composed of Leu671, Met730, and the aliphatic moiety of Lys619. As a potential site for the development of inhibitor specificity, the N9 isopropyl group of C52 points towards Ile650, which is one of the few positions of the nucleotide binding pocket that is divergent among the Axl members (Axl, Mer, and Sky). Although C52 and analogs studied here may not be therapeutically useful because of their potential non-specificity and toxicity, the present structures provide the first view of the Mer kinase domain and will be useful for computational chemistry approaches towards inhibitor discovery and optimization.

High-resolution, complex structures of the Mer kinase domain were determined, showing autoinhibited αC-Glu-out conformations. These structures are the first to be determined from the Mer/Axl/Sky subfamily of receptor tyrosine kinases, and provide a detailed map of the interactions made by residues implicated in retinitis pigmentosa. As this protein is a proto-oncogene and is overexpressed in a large number of tumors, ligands, or chemical probes may help show a potential therapeutic window for Mer kinase inhibition. The inhibited conformation of the N-lobe could be targeted for the discovery and development of anticancer drugs. We identified a preliminary series of ligands that may help address these challenges.

## Figures and Tables

**Table 1:** Data collection, phasing, and refinement statistics.

Dataset	ADP	ANP	C52
PDB code	3BRB	2P0C	3BPR
Space group	P2_1_	P2_1_	P2_1_
Unit cell	53.38 89.88 69.54	52.47 90.06 69.25	70.00 91.70 120.74
	90.00 103.09 90.00	90.00 102.44 90.00	90.00 94.06 90.00
Beamline	CHESS F-1	FR-E	APS 17ID
Wavelength	0.918	1.54178	1
Resolution	25.00–1.90	25.00–2.40	50.00–2.80
Unique reflections	49802	24544	37537
Data redundancy	7.5 (5.2)	3.5 (3.4)	3.3 (2.9)
Completeness⁎⁎	98.3 (88.7)	99.8 (99.7)	99.0 (94.3)
*I*/sig*I*	24.1 (2.4)	21.6 (5.2)	9.1 (2.3)
*R*_sym_⁎⁎⁎	0.09 (0.66)	0.06 (0.29)	0.15 (0.53)

*Refinement*			
Resolution	25–1.9	24.8–2.4	47.3–2.8
Reflections used	47038	23326	35338
All atoms {non-protein atoms}	4502 {303}	4186 {233}	8428 {134}
*R*_work_/*R*_free_⁎⁎⁎⁎	0.19/0.24	0.20/0.27	0.27/0.30
Rmsd bond length	0.013	0.009	0.011
Rmsd bond angle	1.511	1.201	1.242
Figure of merit	0.77	0.82	0.67
Mean B factor	34	39.1	58.4

*Ramachandran plot*			
Favoured	97.9	97.2	95.3
Allowed	2.1	2.8	4.7
Disallowed	0	0	0

Atomic coordinates were deposited in the Protein Data Bank (www.rcsb.org).

⁎⁎ Highest-resolution shell is shown in parentheses.

⁎⁎⁎ *R*_sym_ = 100 × sum(|*I* − 〈*I*〉|)/sum(〈*I*〉), where *I* is the observed intensity and 〈*I*〉 is the average intensity from multiple observations of symmetry-related reflections.

⁎⁎⁎⁎ *R*_free_ value was calculated with 5% of the data.
